# Syndrome of inappropriate secretion of antidiuretic hormone following high dose rate brachytherapy for prostate cancer: a case report

**DOI:** 10.1186/s12894-022-00984-y

**Published:** 2022-03-10

**Authors:** Adeoluwa Ayoola, Quaovi H. Sodji, Sharon Chin, Periklis Panousis, Hilary P. Bagshaw, Mark K. Buyyounouski

**Affiliations:** 1 Stanford University School of Medicine, Stanford, CA USA; 2 Department of Radiation Oncology, Stanford University School of Medicine, 875 Blake Wilbur Drive, Stanford, CA 94305 USA; 3 Department of Anesthesiology, Stanford University School of Medicine, Stanford, CA USA

**Keywords:** High dose rate brachytherapy, Radiotherapy, Prostate cancer, Syndrome of inappropriate antidiuretic hormone secretion, Hyponatremia

## Abstract

**Background:**

The syndrome of inappropriate secretion of antidiuretic hormone is a disorder characterized by the excess release of antidiuretic hormone and can result in hyponatremia. If managed inappropriately, severe hyponatremia can cause seizures, cerebral edema, and even death. There are various known causes of this inappropriate release of antidiuretic hormone, including malignancy, CNS disorders, and disturbances in the hypothalamic-pituitary-renal axis. However, reports of syndrome of inappropriate secretion of antidiuretic hormone after brachytherapy for prostate cancer are exceedingly rare.

**Case presentation:**

We report a case of symptomatic hyponatremia secondary to the inappropriate secretion of antidiuretic hormone after prostate high-dose rate brachytherapy under general anesthesia in a patient with adenocarcinoma of the prostate.

**Conclusions:**

In rare instances, inappropriate secretion of antidiuretic hormone can occur after high-dose rate brachytherapy for prostate cancer. The cause is likely multifactorial, involving pain or discomfort ensuing from the surgical procedure, the general anesthesia or intraoperative drugs administered. However, due to the potential severity of the side effects, timely diagnosis is crucial to ensure prompt, and effective management.

## Background

The syndrome of inappropriate secretion of antidiuretic hormone (SIADH) is a disorder defined by excess release of antidiuretic hormone (ADH), resulting in increased water retention [1]. The Bartter and Schwartz criteria for diagnosing SIADH includes decreased serum osmolality, less than 275 milliosmole (mOsm), increased urine osmolality, greater than 100 mOsm, euvolemia, increased urine sodium, greater than 20 milliequivalent/L (mEq/L), and no other cause for hyponatremia such as diuretics, hypercortisolemia, thyroid dysfunction [2, 3]. Etiologies of SIADH include malignancy, CNS disorders, especially disturbances in the hypothalamic-pituitary-renal axis, pulmonary diseases, drugs, transient physiologic stressors including pain and infection, and surgical procedures [1, 2]. The resulting hypotonic hyponatremia, leads to intracellular water shift and symptoms such as headaches, nausea, vomiting, confusion, and seizures when severe [1, 2, 4].

There are few cases of symptomatic hyponatremia due to SIADH following radiation therapy (RT) in the literature. McDonald et al. reported a case after palliative RT for non-small cell lung cancer, hypothesizing that the SIADH was caused by the release of ADH by dying malignant cells [5]. Similar to the case reported here, Slevin et al. described a case of severe hyponatremia and seizures due to SIADH in a 61-year-old prostate cancer patient after a HDR procedure, hypothesizing that the use of general anesthesia and trauma brought on by the procedure were both contributing factors [6].

Herein, we report a case of symptomatic hyponatremia due to SIADH after prostate HDR brachytherapy.

## Case presentation

A 74-year-old male with a cT1cN0M0, Gleason 4 + 3, and a pre-treatment prostate specific antigen (PSA) of 3.9 ng/mL, NCCN unfavorable intermediate-risk adenocarcinoma of the prostate was prescribed combination therapy with prostate HDR brachytherapy to 15 Gy in 1 fraction followed by pelvic nodes external beam RT to 46 Gy in 23 fractions with neoadjuvant, concurrent and adjuvant ADT for 4 months. His past medical history included asthma, palpitations, hypercholesterolemia, and hypertension. His medications included atenolol, atorvastatin, and leuprolide.

The patient’s HDR brachytherapy procedure was uncomplicated and performed under general anesthesia, our institutional standard. Fifteen needles were inserted transperineally for delivery of the radiation dose. There were no signs of distress or discomfort during the procedure. During the procedure, he received 1.5 L of normal saline and had 0.45 L of urine output. Post procedure, he was transferred to the PACU, where he stayed for ten hours before discharge. While in the PACU, he had no nausea, vomiting or confusion, however he did develop polydipsia. On retrospective chart review, he was found to have ingested a total of 8.250 L of water, consuming approximatively 0.5–1.5 L per hour, but only had 0.425 L of urine output, with a net positive fluid balance of 8.875 L. Prior to discharge, he reported a mild nausea but felt overall well, and met the PACU standard discharge criteria and was sent home. Overnight, following his discharge, he had a new onset of intractable nausea, projectile vomiting, and abdominal discomfort. The next morning, he presented to the emergency department with generalized weakness, dizziness, and lightheadedness. On physical examination, he had dry mucous membranes and further work up revealed hyponatremia with a serum sodium of 120 mEq/L. Preoperative labs including serum sodium obtained a week before the procedure were normal. Serum osmolality, urine osmolality and urine sodium were respectively 250 mEq/L (Normal range 285–310 mEq/L), 712 mEq/L (Normal range 300–900 mEq/L), and 137 mEq/L (Normal range $\ge$ 20 mEq/L).

He was subsequently admitted for acute severe hyponatremia due to SIADH. His sodium was slowly corrected with hypertonic saline for a goal of 6–8 mEq/L per day and he was also placed on fluid restriction, less than 2 L of free water daily. His symptoms including nausea, vomiting, lightheadedness, and dizziness improved by the next day as his serum sodium started to improve and all symptoms resolved by the second hospitalization day. His hospitalization lasted for 2 days and he was discharged with a serum sodium of 130 mEq/L. He was counseled to maintain a 2 L per day fluid restriction and to have close follow-up with his primary care physician for repeat labs. On follow-up labs 2 days after his hospital discharge, his sodium remained within normal limits and his fluid restriction was lifted.

## Discussion and conclusions

The post-operative period carries a high risk of developing SIADH and subsequently hyponatremia which can have severe and potentially fatal complications such as cerebral edema. Chung et al. found that post-operatively, 4% of patients develop hyponatremia secondary to SIADH [7]. SIADH following prostate HDR brachytherapy has been previously reported in one patient who developed severe hyponatremia causing seizures [6]. The etiology of this patient’s SIADH was believed to be multifactorial including general anesthesia and trauma from the catheters’ placement. This case was similar to ours reported here, where the patient also developed SIADH after the same procedure under general anesthesia. While rare with only 2 cases of literature reports, medical practitioners including oncologists should be aware of the potential development of SIADH in the post-brachytherapy setting.

SIADH can occur as a paraneoplastic syndrome in patients with certain malignancies, most commonly small cell carcinoma. Small cell carcinoma, a neuroendocrine tumor, is most commonly diagnosed of the lung; however, this histology is occasionally seen in the prostate. There have been some reports of SIADH diagnosed in small cell prostate cancer patients, as well as patients with adenocarcinoma with neuroendocrine features, unrelated to brachytherapy [8, 9]. In our case, the patient had adenocarcinoma of the prostate without neuroendocrine differentiation, suggesting an etiology other than the histology of his malignancy.

Timely and accurate recognition of SIADH-induced hyponatremia is crucial, as it is a potentially permanent and life-threatening condition and often not managed adequately [2]. Delay in diagnosis can result in longer hospital stays and, in some instances, higher morbidity and mortality [2, 10, 11]. In some instances, the SIADH-induced hyponatremia can be worsened by the presence of polydipsia which is a consequence of the release of ADH under normal physiological conditions. Ways to prevent the development of hyponatremia in case of SIADH involve adequate monitoring of fluid intake and output for early diagnosis and intervention. This includes tracking fluid intake post-operatively, identifying polydipsia and early limitation of fluid intake. In addition to fluid restriction, another step in the management of SIADH includes the intravenous administration of hypertonic fluids such as a 3% hypertonic sodium chloride solution [1]. Isotonic fluids such as normal saline should be avoided as they can worsened the hyponatremia [7, 12, 13]. Although, SIADH-induced hyponatremia can have potentially fatal complications, its rapid correction can lead to osmotic demyelination syndrome (ODS), which carries also a dismal prognosis [1, 4, 11]. As such, the correction of hyponatremia should not exceed 8–12 mEq/L in 24 h to reduce the risk of ODS [1, 14]. In addition to correcting the hyponatremia, identifying and addressing the underlying etiology of such SIADH is ultimately the key aspect of patient management under these circumstances.

Hyponatremia is often diagnosed late or completely missed, which can have deleterious effects on patient outcomes. SIADH is a common etiology of hyponatremia which can be seen in the postoperative or peri-procedural setting. As such, early and adequate intervention can be critical. Despite the fact that RT has not been directly implicated as a cause of SIADH, our report adds to the list of literature, suggesting that HDR prostate brachytherapy is a possible etiology of SIADH. Although, the factors associated with prostate HDR brachytherapy contributing to SIADH may be multifactorial such as procedural trauma or general anesthesia, early diagnosis through adequate charting of fluid intake and output and subsequent intervention is key to preventing harmful short and long-term side effects.

## Data Availability

Data sharing is not applicable to this article as no datasets were generated or analyzed during the current study.

## Abbreviations

ADH: Antidiuretic Hormone
ADT: Androgen deprivation therapy
HDR: High dose rate
mOsm: Milliosmole
mEq/L: Milliequivalent/L
NCCN: National comprehensive cancer network
ODS: Osmotic demyelination syndrome
PACU: Post-anesthesia care unit
PSA: Prostate specific antigen
RT: Radiation therapy
SIADH: Syndrome of inappropriate secretion of antidiuretic hormone

## References

[1] Ellison DH, Berl T (2007). The syndrome of inappropriate antidiuresis. N Engl J Med.

[2] Hoorn EJ, van der Lubbe N, Zietse R. SIADH and hyponatraemia: why does it matter? NDT Plus 2009;2: iii5–iii11. 10.1093/ndtplus/sfp153 PMC2762826 19881934

[3] Schwartz WB, Bennett W, Curelop S (1957). A syndrome of renal sodium loss and hyponatremia probably resulting from inappropriate secretion of antidiuretic hormone. Am J Med.

[4] Adrogué HJ, Madias NE (2000). Hyponatremia. N Engl J Med.

[5] McDonald P, Lane C, Rojas GE (2012). Syndrome of inappropriate anti-diuretic hormone in non-small cell lung carcinoma: a case report. Ecancermedicalscience.

[6] Slevin F, Rodda S, Bosomworth M (2014). Hyponatraemic seizures following prostate brachytherapy. J Radiother Pract.

[7] Chung HM, Kluge R, Schrier RW (1986). Postoperative hyponatremia A prospective study. Arch Intern Med.

[8] Fiordoliva I, Marcantognini G, Rinaldi S, Cimadamore A, Montironi R, Berardi R. Syndrome of inappropriate antidiuresis in prostate adenocarcinoma with neuroendocrine differentiation: a case report and literature review. J Cancer Metastasis Treat 2019; 5.

[9] Fukasawa M, Sawada N, Shimura H (2017). Successful radiotherapy for advanced small cell carcinoma of the prostate with syndrome of inappropriate secretion of antidiuretic hormone. Urol Case Rep.

[10] Gill G, Huda B, Boyd A (2006). Characteristics and mortality of severe hyponatraemia – a hospital-based study. Clin Endocrinol.

[11] Peri A (2019). Morbidity and mortality of hyponatremia. Front Horm Res.

[12] Steele A, Gowrishankar M, Abrahamson S, Mazer CD, Feldman RD, Halperin ML (1997). Postoperative hyponatremia despite near-isotonic saline infusion: a phenomenon of desalination. Ann Intern Med.

[13] Moritz ML, Ayus JC (2007). Hospital-acquired hyponatremia–why are hypotonic parenteral fluids still being used?. Nat Clin Pract Nephrol.

[14] Janicic N, Verbalis JG (2003). Evaluation and management of hypo-osmolality in hospitalized patients. Endocrinol Metab Clin North Am.

